# Molecular genetic analysis of Rubinstein–Taybi syndrome in Russian patients

**DOI:** 10.3389/fgene.2025.1516565

**Published:** 2025-01-31

**Authors:** Olga R. Ismagilova, Tagui A. Adyan, Tatiana S. Beskorovainaya, Alexander V. Polyakov

**Affiliations:** ^1^ DNA-Diagnostics Laboratory, Federal State Budgetary Scientific Institution, Research Centre for Medical Genetics (RCMG), Moscow, Russia; ^2^ Department of General and Medical Genetics, Faculty of Biomedical Sciences, Pirogov Russian National Research Medical University, Moscow, Russia

**Keywords:** multiple congenital anomaly syndrome, Rubinstein–Taybi syndrome, CREBBP, EP300, next-generation sequencing, multiplex ligation-dependent probe amplification

## Abstract

**Introduction:**

Rubinstein–Taybi syndrome (RSTS) is one of the many forms of syndromic intellectual disability, occurring in the population with a frequency of 1: 100–125 thousand newborns. The specific phenotype of patients enables the so-called “portrait” diagnosis of classical cases of RSTS, followed by the analysis of the *CREBBP* and EP300 genes, whose association with RSTS has been confirmed. Nevertheless, for approximately half of the patients in various cohorts, the diagnosis cannot be confirmed.

**Methods:**

In this paper we present the results of a study of 158 Russian patients referred for molecular diagnosis of RSTS using multiplex ligation-dependent probe amplification (MLPA) and next-generation sequencing (NGS).

**Results:**

Pathogenic and likely pathogenic variants were identified in 67 patients (42.4%), of which 62 (39%) were in *CREBBP* and 4 cases (2%)—in EP300. In one case, a known pathogenic variant in *SRCAP*, associated with Floating–Harbor syndrome (FHS), which is phenotypically similar to RSTS, was also identified; therefore, the possibilities and prospects for differential diagnosis were considered.

## 1 Introduction

Rubinstein–Taybi syndrome (RSTS) is a multisystemic pathology characterized by intellectual disability and delayed postnatal physical development, accompanied by a complex of phenotypic signs, such as broad thumbs and toes, microcephaly, and facial dysmorphisms such as downslanted palpebral fissures, high-arched eyebrows, a broad and “beaked” nasal bridge, a columella below the alae nasi, a narrow and high-arched palate, dental abnormalities, and a “grimacing” smile. This is the classic portrait of RSTS. However, after discovering a second gene linked to the syndrome–*EP300*, it became clear that the phenotypic manifestations vary widely. Currently, there are two genetic variants of RSTS: type 1, associated with the *CREBBP* gene, was first identified as the cause of RSTS in 1995 and was cloned 2 years later; type 2 is associated with *EP300*, with the first cases with variants described in 2005 (Petrif et al., 1995; Roelfsema et al., 2005). Characterized by a high degree of homology, both genes have the same domain structure and encode proteins with more than 70% similarity in their amino acid sequences. The protein products of these genes, CBP (CREB-binding protein, for short - CBP) and p300 (E1A-associated protein), are involved in the regulation of transcription and the function of many regulatory proteins in the cell. For a long time, sequencing these genes was difficult due to their large size, but since their association with RSTS was discovered, the development of new methods, such as next-generation sequencing (NGS) has facilitated ongoing efforts to unravel the molecular mechanisms of pathogenesis.

The purpose of this study was to identify the spectrum of mutations in genes responsible for RSTS in Russian patients. This article presents the results from a cohort of 158 probands referred to the Research Center for Medical Genetics (RCMG). The methods included searching for large deletions and duplications in *CREBBP* and *EP300* by multiplex ligation-dependent probe amplification (MLPA). In cases where large deletions or duplications were not found, patients underwent a sequence analysis of the coding regions of *CREBBP* and *EP300* using NGS.

## 2 Materials and methods

### 2.1 Patients

A cohort of 158 patients with a clinically suspected diagnosis of RSTS was enrolled from 2013 to 2022 based on the RCMG. The analyzed sample included 91 boys and 67 girls, with a median age of 4 years. The initial testing was organized as part of routine genetic diagnostics upon request, and our cohort included all referred cases, regardless of the phenotype. Some patients were examined in the clinical unit of the RCMG, while others were referred by doctors from various regions of Russia. Not all referred samples had data on clinical manifestations, and the volume of available clinical data in the majority of cases was insufficiently structured to search for possible genotypic–phenotypic correlations. Therefore, their assessment was not carried out due to potential cohort bias. Available clinical information on patients with identified variants presented in this study can be found in Supplementary Tables S1, S2. Ethical approval was granted by the Ethics Committee of the RCMG (protocol 6/3 of 19 April 2021). Informed consent for using and publishing the anonymized results was obtained from all patients or their legal representatives. DNA was purified from whole blood in EDTA using the Wizard^®^ Genomic DNA Purification Kit (Promega, United States), according to the manufacturer’s protocol.

### 2.2 Research algorithm

The study was organized based on the algorithm proposed by Hennekam (2006). Initially, all patients were screened for large deletions and duplications in *CREBBP* and *EP300* using the MLPA kit (MRC-Holland, The Netherlands). Subsequently, DNA samples from patients with no changes detected in the first stage were examined using the NGS method with a custom panel that included coding exons of the *CREBBP* and *EP300* genes to search for point mutations and small deletions/insertions undetectable by MLPA.

### 2.3 MLPA analysis

To identify large deletions and duplications in *CREBBP* and *EP300*, the SALSA MLPA Probemix P313-B3 CREBBP Kit was used, which contains a mixture of probes for exons 1–31 of *CREBBP* (NM_004380.3) and exons 1, 4, and 12 of *EP300* (NM_001429.4). The kit includes two probes flanked exons 1–3 of the *CREBBP* gene on both sides, which makes it possible to determine copy number changes without additional tests. If a decrease in signal intensity was detected for only one of the remaining exons, marked by a single pair of probes, that exon was also examined by Sanger sequencing to exclude small variants that might disrupt probe annealing at the site.

### 2.4 NGS analysis of *CREBBP*, *EP300*, and *SRCAP*

For further investigation, a panel including coding regions and exon–intron junctions of *CREBBP*, *EP300*, and *SRCAP* was developed, with a total length of 28,967 bp, distributed into 213 amplicons. In addition to the two main genes associated with RSTS, this panel also included *SRCAP*, which is linked to the development of Floating–Harbor syndrome (FHS) and is phenotypically similar to RSTS (Spena et al., 2015). Library preparation for subsequent sequencing on an Ion S5 (Life Technologies, United States) was performed using the Ion AmpliSeq™ Library Kit 2.0 reagents (Thermo Fisher Scientific, United States) in accordance with the manufacturer’s protocol. Verification of the identified variants by NGS was carried out by the polymerase chain reaction (PCR) followed by Sanger sequencing using an ABI PRISM 3500XL Genetic Analyzer (Applied Biosystems, United States). Clinical significance was evaluated using the “Guidelines for interpretation of human DNA sequence obtained with mass parallel sequencing (MPS)” (Ryzhkova et al., 2019). This guideline is an adapted version of the ACMG guidelines for the interpretation of sequence variants (Richards et al., 2015) and uses a similar approach to determine variant pathogenicity. The PM1 criterion (mutational hotspot and/or a critical and well-established functional domain in which the variant is located) was not used in the classification of variants in this study. Since the main type of pathogenic variants for RSTS leads to haploinsufficiency or the synthesis of truncated proteins, missense variants that have not been previously described in the literature were classified as variants of uncertain clinical significance (VoUSs).

## 3 Results

Using the consecutive search of large intragenic rearrangements and NGS of three selected genes, pathogenic and likely pathogenic variants were identified in 67 out of 158 patients examined (42.4%). In 91 patients (57.6%), the molecular genetic diagnosis could not be confirmed after two stages of the study. However, of these 91 cases, variants of uncertain clinical significance were identified in five patients (3.2%). Additional segregation analysis in their families could enable the reclassification of the clinical significance of these variants.

**FIGURE 1:**
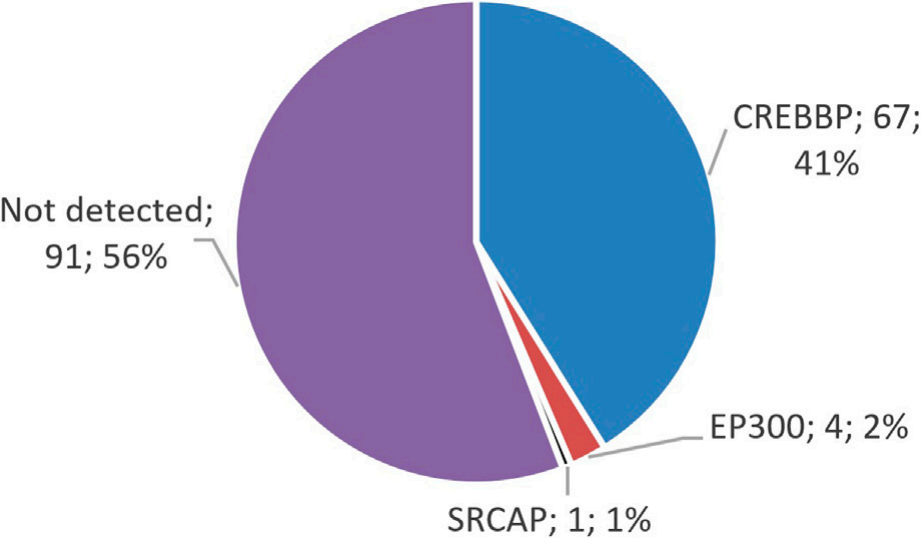
Distribution of identified variants in the genes *CREBBP*, *EP300*, and *SRCAP*. The majority of the identified variants in patients with suspected RSTS are located in *CREBBP*, which is linked to RSTS-1.

The distribution of variants in genes shows that the majority of the identified changes—62 variants—were found in *CREBBP*; 4 variants were found in *EP300*, and 1 previously described variant was found in *SRCAP* (Figure 1). Clinical data for this patient were not available, but this finding supports the expediency of differential diagnosis among RSTS-like phenotypes.

Large deletions and duplications leading to a complete loss of one copy of the gene or a critical change in the gene structure were identified only in *CREBBP* (Table 1). For this gene, their share was 31% (19 cases) relative to the entire examined cohort—12%. Some samples showed a repeating pattern in the results of MLPA imaging (1 exon, 4–31 exons, and the entire *CREBBP* gene); however, in this work, the exact boundaries of the rearrangements were not established and may be different in each case. Neither deletions nor duplications in *EP300* were detected, but the incomplete coverage of this gene by the probes available in the MLPA kit used does not allow us to completely exclude the presence of such changes in uncovered regions. In addition, in two patients, two different likely pathogenic variants were identified in *CREBBP*, both leading to the premature termination of transcription due to a decrease in signal intensity for only one exon, which was subsequently examined by Sanger sequencing. The diagnostic search for these patients was completed at this stage.

**TABLE 1:** Deletions and duplications found in the RTS cohort.

*CREBBP (NC_000016.11 (NM_* 004380.3*)*			
Number of patients	Involved exon	Variant	Domain
Deletion			
2	Exon 1	c. (?_-1)_(85 + 1_86–1)	
1	Exons 1–17	c. (?_-1)_(3,369 + 1_3370–1)	TAZ1–BRD
1	Exon 16	c. (3,060 + 1_3061–1)_(3,250 + 1_3251–1)	
1	Exons 22–28	c. (3,836 + 1_3837–1)_(4,728 + 1_4729–1)	PHD–HAT
1	Exons 3–31	c. (798 + 1_799–1)_(*1_?)	TAZ1–TAZ2
3	Exons 4–31	c. (975 + 1_976–1)_(*1_?)	TAZ1–TAZ2
1	Exons 30–31	c. (4,890 + 1_4891–1)_(*1_?)	HAT–TAZ2
1	Exons 27–31	c. (4,394 + 1_4395–1)_(*1_?)	HAT–TAZ2
5	Exons 1–31	c. (?_–1)_(*1_?)	Whole gene
Duplication			
1	Exons 12–15	c. (2,158 + 1_2159–1)_(3,060 + 1_3061–1)	
1	Exons 5–28	c. (1,216 + 1_1217–1)_(4,728 + 1_4729–1)	TAZ1–HAT
1	Exons 21–30	c. (3,779 + 1_3780–1)_(5,172 + 1_5173–1)	PHD–ZZ

**FIGURE 2:**
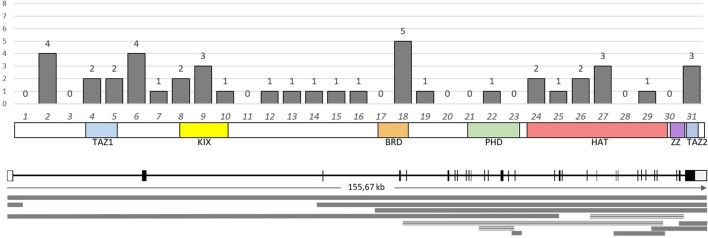
Distribution and number of identified variants in the exons of the *CREBBP* gene. Below the graph is a schematic representation of the domain structure of the CBP protein according to the exonic structure. The lower part of the image presents a diagram of the distribution of large deletions (solid line) and duplications (triple line) identified in this study.

**FIGURE 3:**
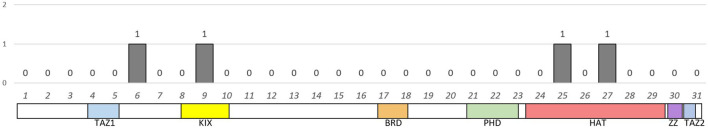
Distribution and number of identified variants in the exons of the *EP300* gene. Below the graph is a schematic representation of the domain structure of the p300 protein according to the exonic structure.

Pathogenic and likely pathogenic point variants in this study were identified using NGS in a total of 48 patients (30.37%). In 43 cases (27.2%), *CREBBP* was involved; in four cases, *EP300* was found; and one variant was found in *SRCAP*. Parental DNA samples were available for only five cases, in which the *de novo* emergence of the variants was confirmed. The vast majority of cases of RSTS represent the first occurrence in a family, but parental testing is important, at least for the reclassification of variants of uncertain clinical significance, such as missense variants, deletions, or insertions without frameshift and any changes outside the canonical splice sites. Table 2 presents all pathogenic and likely pathogenic variants identified in this work. Figures 2, 3 show the distribution of variants in *CREBBP* and *EP300* by exons, according to the domain structure of the encoded protein.

**TABLE 2:** Pathogenic and likely pathogenic variants in *CREBBP*, *EP300*, and *SRCAP* identified in this work.

Number of patients	Variant	Domain	ACMG classification	Inheritance	Source/HGMD ID
*CREBBP (NM_* 004380.3*)*					
Frameshift variant					
1	c.124del, p. (Asp42fs)		PVS1, PS2, and PM2	*de novo*	This work
1	c.365del, p. (Pro122fs)		PVS1 and PM2	Not tested	This work
1	c.1293_1296del, p. (Pro432fs)	TAZ1	PVS1 and PM2	Not tested	This work
1	c.1669dup, p. (Ala557fs)		PVS1 and PM2	Not tested	This work
1	c.1717del, p. (Thr573fs)	KIX	PVS1 and PM2	Not tested	This work
1	c.1890del, p. (Ala631fs)	KIX	PVS1 and PM2	Not tested	This work
1	c.1894dup, p. (Tyr632fs)	KIX	PVS1 and PM2	Not tested	This work
1	c.1911del, p. (Asp639fs)	KIX	PVS1 and PM2	Not tested	This work
1	c.2053_2054dup, p. (Leu685fs)		PVS1 and PM2	Not tested	This work
1	c.2429dup,p. (Met810fs)		PVS1 and PM2	Not tested	This work
1	c.2663del, p. (P888fs)		PVS1 and PM2	Not tested	This work
1	c.2898del, p. (Ser967fs)		PVS1 and PM2	Not tested	This work
1	c.3392dup, p. (Asn1131fs)	BRD	PVS1, PS2, and PM2	*de novo*	This work
1	c.3544_3554del, (p.Ala1182fs)	BRD	PVS1 and PM2	Not tested	This work
1	c.4074del, p. (Phe1358fs)	HAT	PVS1 and PM2	Not tested	This work
1	c.4129_4132dup, p. (Arg1378fs)	HAT	PVS1, PS2, and PM2	*de novo*	This work
1	c.4729_4733del, p. (Gly1577fs)	HAT	PVS1 and PM2	Not tested	This work
1	c.5757_5769dup, p. (Val1924fs)		PVS1 and PM2	Not tested	This work
Nonsense variant					
1	c.316C>T, p. (Gln106*)		PVS1 and PM2	Not tested	This work
1	c.445C>T, p. (Gln149*)		PVS1 and PM2	Not tested	This work
1	c.733C>T, p. (Gln245*)		PVS1 and PM2	Not tested	This work
1	c.1063C>T, p. (Gln355*)	TAZ1	PVS1 and PM2	Not tested	This work
1	c.1114C>T, p. (Gln372*)	TAZ1	PVS1 and PM2	Not tested	This work
1	c.1270C>T, p. (Arg424*)	TAZ1	Previously described as pathogenic	Not tested	CM053181, PMID: 16021471 and 32827181
2	c.1447C>T, p. (Arg483*)		Previously described as pathogenic	Not tested	CM1918549, PMID: 31566936
1	c.1522C>T, p. (Gln508*)		PVS1 and PM2	Not tested	This work
1	c.2218G>T, p. (Gly740*)		PVS1 and PM2	Not tested	This work
1	c.3441C>A, p. (Tyr1147*)	BRD	PVS1 and PM2	Not tested	This work
1	c.3690T>G, p. (Tyr1230*)		Previously described as pathogenic	Not tested	CM2023000, PMID: 32827181
1	c.3911C>A, p. (Ser1304*)	PHD	PVS1 and PM2	Not tested	This work
1	c.4380T>A, p. (Tyr1460*)	HAT	PVS1 and PM2	Not tested	This work
Splicing variant					
1	c.1574–2A>G		PVS1 and PM2	Not tested	This work
1	c.1824-1G>C		PVS1 and PM2	Not tested	This work
1	c.3242_3250+6del		PVS1 and PM2	Not tested	This work
1	c.3609 + 1G>A		Previously described as pathogenic	Not tested	CS2057719, PMID: 33057194 and 35982159
1	c.3610−1G>C		PVS1 and PM2	Not tested	This work
1	c.3698 + 5G>A		PS1, PS2, and PM2	*de novo*	This work
1	c.4279_4280+1del		PVS1 and PM2	Not tested	This work
1	c.4560 + 1G>A		PVS1 and PM2	Not tested	This work
Missense variant					
1	c.4340C>T, p. (Thr1447Ile)	HAT	Previously described as pathogenic	Not tested	CM050568, PMID: 15706485
2	c.4439A>G, p. (Asp1480Gly)	HAT	Previously described as pathogenic	Not tested	CM1925364 PMID: 31216405, 32827181, and 33057194
*EP300 (NM_001429.4)*					
Frameshift variant					
1	c.4043dup, p. (Met1349fs)	HAT	PVS1 and PM2	Not tested	This work
Nonsense variant					
1	c.1459C>T, p. (Gln487*)		PVS1 and PM2	Not tested	This work
1	c.4384C>T, p. (Arg1462*)	HAT	Previously described as pathogenic	Not tested	CM2119308, PMID: 34427995 and 35616356
Splicing variant					
1	c.1878_1878+1del	KIX	PVS1 and PM2	Not tested	This work
*SRCAP (NM_006662.3)*					
1	c.7303C>T, p. (Arg2435*)		Previously described as pathogenic	Not tested	CM121173, PMID: 22265015 and 38230957

Six variants in *CREBBP* and one variant in *EP300* identified in this cohort were previously described in the literature in patients with RSTS. Two of these variants—the nonsense variant c.1447C > T p (Arg483*) and the missense substitution c.4439A > G, p. (Asp1480Gly) in *CREBBP*—were each found in two patients from two unrelated families (Cross et al., 2020; Coupry et al., 2002).

The sample in this study is highly heterogeneous, making it impossible to establish genotype–phenotype correlations. Clinical information, including the phenotypic characteristics of the patients, was available for only 24 probands with identified pathogenic and likely pathogenic variants but was insufficiently structured for proper analysis. All patients were sent directly for confirmation or exclusion of the diagnosis, and we assume that each patient demonstrated some of the main clinical features of RSTS.

**FIGURE 4:**
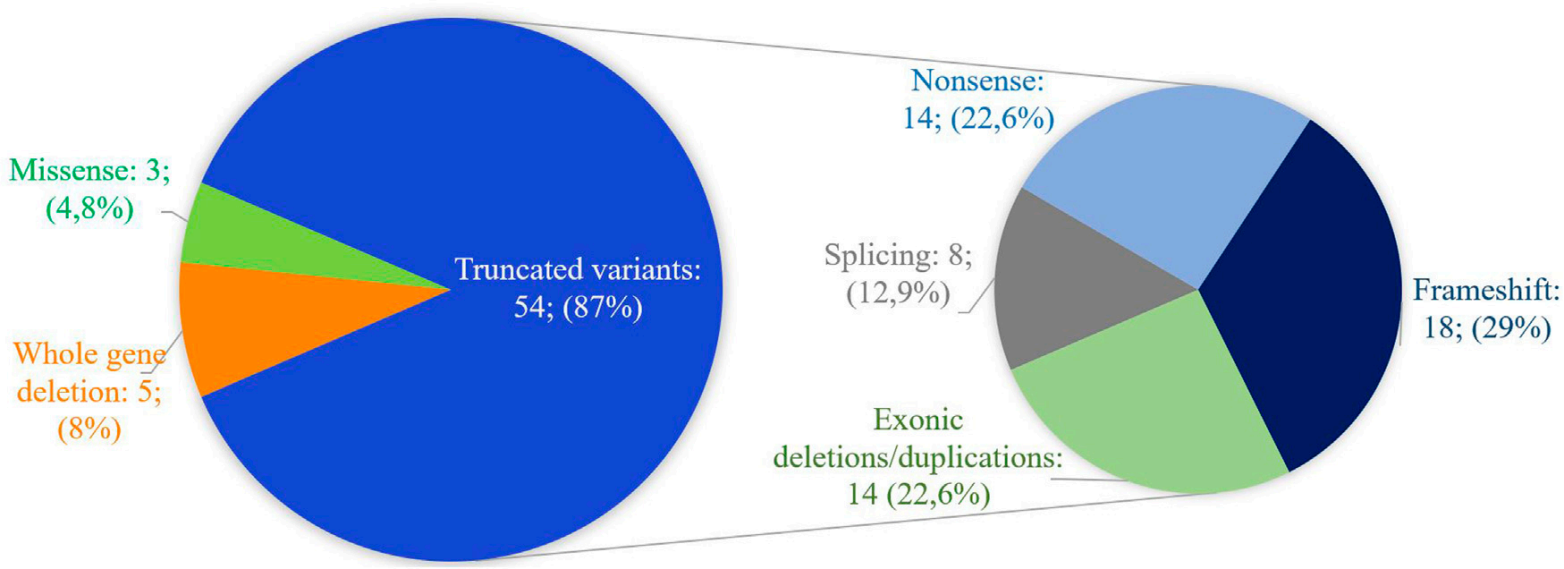
Spectrum of pathogenic and likely pathogenic variants in *CREBBP* identified in this work. The majority of variants lead to the presumed synthesis of a truncated protein or the absence of product synthesis from one of the alleles.

Figure 4 shows the range of pathogenic and likely pathogenic variants in *CREBBP* identified in this study.

In addition, in the studied cohort, four VoUSs in *CREBBP* were identified (Table 3)—two missense variants in exons 19 and 31, one variant that presumably affects splicing due to a single nucleotide substitution at the +5 position of intron 9, and an in-frame deletion of 15 bp. *In silico* predictors of variant impact suggested that each of these variants was pathogenic. Moreover, three variants that change the amino acid sequence itself (missense and in-frame deletion) are located in functionally important domains; therefore, they could not be confidently excluded from the list of variants that may affect the formation of the RSTS phenotype. One missense variant in *EP300*, located in the PHD domain, was classified as a VoUS because it met only the PM2 and PP3 criteria (Ryzhkova et al., 2019).

**TABLE 3:** VoUSs in CREBBP and EP300 identified in this work.

Variant	Domain	ACMG classification	Inheritance	Source/HGMD ID
*CREBBP*				
Splicing				
c.1941 + 5G>A	KIX	PM2 and PP3	Not tested	This work
Missense				
c.3698G>C, p. (Arg1233Thr)		PM2 and PP3	Not tested	This work
c.5486A>T, p. (His1829Leu)	TAZ2	PM2 and PP3	Not tested	This work
Inframe				
c.4240_4254del p. (Val1414_Gly1418del)	HAT	PM2 and PP3	Not tested	This work
*EP300*				
c.3749G>A, p. (Cys1250Tyr)	PHD	PM2 and PP3	Not tested	This work

## 4 Discussion

In the cohort of patients with RSTS studied in this work, the proportion of identified pathogenic and likely pathogenic variants was 43.7%. Currently, according to worldwide studies of various cohorts of patients with RSTS, the detection of the cause of the disease ranges from 37% to 75% (Coupry et al., 2002—47.6%; Bartsch et al., 2005—56% in patients with classical RSTS and 25% in those with a putative but “incomplete” phenotype; Bentivegna et al., 2006—61.3%; Schorry et al., 2008—56%, Wincent et al., 2016—70.6%; and Cross et al., 2020—37%) (Cross et al., 2020; Coupry et al., 2002; Bartsch et al., 2005; Bentivegna et al., 2006; Schorry et al., 2008; Wincent et al., 2016). These highly variable results are likely due to differences in cohort size, selection criteria, and the detected genetic and phenotypic heterogeneity of the syndrome.

The lack of structured clinical data in this study likely contributed to the relatively low detection rate compared to that in previously reported global data based on studies of clinically well-described cohorts of patients with classical RSTS. In patients without an identified cause, variants in large non-coding regions of genes, along with large rearrangements (for example, translocations) or deletions in regions not covered by the MLPA set (for example, in the gene *EP300*), cannot be excluded. Thorough clinical analysis and description of the phenotype could increase the proportion of cases with a confirmed diagnosis. Identifying groups of key and less specific features, while counting their occurrences could probably allow for a more confident distinction between classical RSTS with a clear phenotype and less typical cases that still exhibit certain manifestations. Recently, an international consensus statement on RSTS was published, in which experts proposed cardinal and supportive diagnostic criteria and a key to assessing their combination in a patient (Lacombe et al., 2024). Despite the possible variability of the phenotype and the fact that some cases of RSTS remain without molecular–genetic confirmation (including patients with identified VoUSs), a clinical diagnosis is necessary to build an optimal diagnostic algorithm that provides genetic counseling to patients’ families and manage patients throughout their lives. VoUSs also require a reassessment of the clinical hypothesis or additional studies to establish their causality for the specific patient. The expansion of diagnostic capabilities, along with the search for potential therapeutic methods, requires further study of RSTS in global practice.

Attempts have been made to identify genotype–phenotype correlations for RSTS, but contradictory data exist regarding the impact on phenotype of various mutations located in different regions of the gene and deletions of various ranges. For example, variants in the proximal part of *CREBBP* lead to the formation of a premature stop codon that may not be associated with severe intellectual disability, which is usually one of the key features of the syndrome. On the contrary, variants in exon 31 of *EP300* lead to a severe form of classic RSTS developmental defects (Pérez‐Grijalba et al., 2019; Cohen et al., 2020). In the same study, the authors described splice site disruption in the HAT domain in a patient with intact intelligence and an unremarkable phenotype with severe growth retardation as the main clinical manifestation. Despite such contradictions, in our cohort, the predominant localization of missense variants in conserved regions of functional domains is consistent with that in the literature, which suggests that the disruption of the main functions of the protein is a key mechanism in the pathogenesis of RSTS. Missense variants in exons 30 and 31 of *CREBBP* and *EP300* were recently linked to Menke–Hennekam syndrome (MHS) in patients with an uncertain diagnosis before whole exome sequencing (Menke et al., 2016). All variants described in MHS affect the amino acids in the TAZ2 and ZZ domains. However, in the same region, a large number of missense variants have been reported in patients with classical RSTS, although phenotypically classical forms of these syndromes show visible differences. The reason for this heterogeneity has not yet been established; it is assumed that a disruption of protein–protein interactions occurs, which is probably different from the effects of the variants that lead to the development of RSTS (Banka et al., 2019). The majority of the described pathogenic variants in RSTS are still associated with the premature termination of translation, resulting in the synthesis of a shortened protein or with haploinsufficiency due to early termination or deletion of the genetic material.

The variability in the clinical manifestations of RSTS, along with its overlap with other chromatinopathy syndromes, highlights the challenges of differential diagnosis and underscores the need for larger-scale studies involving patients. Potentially, it can also expand our understanding of the molecular mechanisms underlying the development of pathology. Currently, there is limited information in the literature regarding the results of whole exome and whole genome sequencing in patients with suspected RSTS without identified changes in *CREBBP* and *EP300*. Several studies have described cross-sectional changes between Rubinstein–Taybi, Wiedemann–Steiner, Coffin–Siris, Kabuki, Bohring–Opitz, and Cornelia de Lange syndromes (Enomoto et al., 2022; Negri et al., 2019; Di Fede et al., 2020; Woods et al., 2014; Cucco et al., 2020). The aforementioned nosologies and RSTS are clinically characterized by common features such as delayed physical development, intellectual disability, limb anomalies, and, sometimes, partially similar dysmorphic facial features. Each of these syndromes is associated with genes involved in the epigenetic regulation of cellular processes at different levels. The shared pathogenic effect, characterized by a shift in the equilibrium toward a more closed state of chromatin and changes in the expression levels of regulated genes, can manifest in overlapping clinical signs (Bjornsson, 2015). For example, the product of the *KMT2A* gene is a transcriptional activator with methyltransferase activity targeting the fourth lysine of the H3 histone subunit of the nucleosome (H3K4). Pathogenic variants in *KMT2A* are associated with the clinical manifestations of Wiedemann–Steiner syndrome (OMIM:605130). KMT2D activates transcription through trimethylation of the same amino acid, H3K4, and the disruption of its function is associated with Kabuki syndrome type 1 (OMIM:147920). *ASXL1* encodes a Polycomb-group enhancer protein, and it is presumed to play a role in the differential enhancement of transcription of some genes while weakening that of others involved in embryonic development; mutations in this gene have been identified in Bohring–Opitz syndrome (OMIM:605039). The SRCAP protein is involved in chromatin remodeling and is one of the components that initiates CREB-mediated transcription by activating CBP (OMIM:136140). In each of the described cases, follow-up clinical examination reveals the signs of these syndromes but not of RSTS: contractures and exophthalmos in the case of *ASXL1* mutation and eversion of the lateral third of the lower eyelid with mutations in KMT2D, despite the predominance of features initially suggesting *RSTS*. The only pathogenic variant we identified in *SRCAP*, which is typically associated with Floating–Harbor syndrome, highlights the possibility that similar phenotypes can develop for genes that are part of the same regulatory network. The described cases of this type remain sporadic and usually include patients in whom the initially presumed diagnosis was not exactly confirmed, and therefore, the scope of the search was expanded. At the same time, the strategy of initial whole exome sequencing of patients with the RSTS phenotype is more likely to detect variants in two main genes: *CREBBP* and *EP300* (Yu et al., 2019; Lee et al., 2022).

## 5 Conclusion

The evaluation of patients should include different methods that complement each other’s limitations. Clinical evaluation also plays an important role in formulating the fastest and most effective diagnostic algorithm when a diagnosis can be phenotypically suggested. Despite the available research methods, there are still a large number of cases of RSTS in which the molecular defect cannot be identified. These patients undoubtedly require further study, which may help not only in making an accurate diagnosis but also in the timely recognition and prevention of possible syndrome-specific complications.

## Data Availability

The datasets presented in this article are not readily available because due to patient privacy and ethical restrictions. Requests to access the datasets should be directed to the corresponding author (OI, ismolga.mg@gmail.com).
