# Supplementary material for: Molecular genetic analysis of Rubinstein–Taybi syndrome in Russian patients
Source: Front Genet. 2025 Jan 31;16:1516565. doi: 10.3389/fgene.2025.1516565 (PMC11825781; doi:10.3389/fgene.2025.1516565)
Supplement: Supplementary file 1 [file DataSheet1.pdf]

Supplementary Material

Table S1. Available clinical data of patients with identified deletions and duplications of *CREBBP*.

Known clinical data have been grouped according to the algorithm proposed in the first international consensus statement for RSTS.

Involved exons	Variant	Domain	Referral age	Phenotype data		
				Cardinal	Supportive	Other features
CREBBP (NC_000016.11 (NM_ 004380.3)						
**Deletions**						
exon 1	c.(?_-1)_(85+1_86-1)		0	No data	No data	Query Rubinstein–Taybi syndrome
exon 1	c.(?_-1)_(85+1_86-1)		1	No data	No data	Query Rubinstein–Taybi syndrome
exones 1-17	c.(?_-1)_(3369+1_3370-1)	TAZ1 – BRD	5,4	No data	No data	Query Rubinstein–Taybi syndrome
exon 16	c.(3060+1_3061-1)_(3250+1_3251-1)		0	Microcephaly, columella below alae nasi, broad thumbs and halluces	No data	Prominent forehead, low-set ears, microstomia, short neck, gastroesophageal reflux, diaphragmatic hernia
exones 22-28	c.(3836+1_3837-1)_(4728+1_4729-1)	PHD – HAT	3,5	No data	No data	Query Rubinstein–Taybi syndrome
exones 3-31	c.(798+1_799-1)_(*1_?)	TAZ1 – TAZ2	1,4	No data	No data	Query Rubinstein–Taybi syndrome
exones 4-31	c.(975+1_976-1)_(*1_?)	TAZ1 – TAZ2	6,6	No data	No data	Query Rubinstein–Taybi syndrome
exones 4-31	c.(975+1_976-1)_(*1_?)	TAZ1 – TAZ2	1	Microcephaly, downslanted palpebral fissures, convex nasal ridge, highly arched palate, broad thumbs	No data	Facial hemangioma, microstomia, brachydactyly, thin lips

				and halluces, mild intellectual disability		
exones 4-31	c.(975+1_976-1)_(*1_?)	TAZ1 – TAZ2	3,4	No data	No data	Query Rubinstein–Taybi syndrome
exones 30-31	c.(4890+1_4891-1)_(*1_?)	HAT – TAZ2	4,8	No data	No data	Query Rubinstein–Taybi syndrome
exones 27-31	c.(4394+1_4395-1)_(*1_?)	HAT – TAZ2	1,3	No data	No data	Query Rubinstein–Taybi syndrome
exones 1-31	c.(?_-1)_(*1_?)	Whole gene	2,8	No data	No data	Query Rubinstein–Taybi syndrome
exones 1-31	c.(?_-1)_(*1_?)	Whole gene	11,3	No data	No data	Query Rubinstein–Taybi syndrome
exones 1-31	c.(?_-1)_(*1_?)	Whole gene	1,2	No data	No data	Query Rubinstein–Taybi syndrome
exones 1-31	c.(?_-1)_(*1_?)	Whole gene	1,5	Columella below alae nasi, highly arched palate, broad distal phalanx of fingers and toes, Intellectual disability	Hypertrichosis	Strabismus, low-set displastic ears, short neck, urinary tract anomalies (congenital megaureter, renal diverticulum)
exones 1-31	c.(?_-1)_(*1_?)	Whole gene	4,1	Downslanted palpebral fissures, broad thumbs and halluces, Intellectual disability, postnatal growth retardation	No data	Ptosis, brachidactyly, deformed thorax, urinary tract anomalies (congenital megaureter, non-functional right kidney)
**Duplications**						
exones 12-15	c.(2158+1_2159-1)_(3060+1_3061-1)		12,3	No data	No data	Query Rubinstein–Taybi syndrome
exones 5-28	c.(1216+1_1217-1)_(4728+1_4729-1)	TAZ1 – HAT	1,6	No data	No data	Query Rubinstein–Taybi syndrome
exones 21-30	c.(3779+1_3780-1)_(5172+1_5173-1)	PHD – ZZ	2,3	Downslanted palpebral fissures, convex nasal ridge	No data	Strabismus, low-set ears

Table S2. Available clinical data of patients with identified variants in *CREBBP*, *EP300* and *SRCAP* using NGS.

Variant	Domain	ACMG classification	Inheritance	Referral age	Phenotype data			Source / HGMD ID
					Cardinal	Supportive	Other features	
					CREBBP (NM_ 004380.3)			
					**Frameshift variants**			
c.124del, p.(Asp42fs)		PVS1, PS2, PM2	de novo	1,9	Downslanted palpebral fissures, broad thumbs and halluces, Intellectual disability, postnatal growth retardation	No data	Strabismus, prominent forehead, facial hemangioma, dysplastic ears, 2-3 finger cutaneous syndactyly and transverse palmar crease of the left hand, ventricular noncompaction, cryptorchidism	This work
c.365del, p.(Pro122fs)		PVS1, PM2	not tested	0,8	No data	No data	Query Rubinstein–Taybi syndrome	This work
c.1293_1296del, p.(Pro432fs)	TAZ1	PVS1, PM2	not tested	1,4	No data	No data	Query Rubinstein–Taybi syndrome	This work

c.1669dup, p.(Ala557fs)		PVS1, PM2	not tested	1.7	No data	No data	Query Rubinstein–Taybi syndrome	This work
c.1717del, p.(Thr573fs)	KIX	PVS1, PM2	not tested	0,9	No data	No data	Query Rubinstein–Taybi syndrome	This work
c.1890del, p.(Ala631fs)	KIX	PVS1, PM2	not tested	4,3	No data	No data	Query Rubinstein–Taybi syndrome	This work
c.1894dup, p.(Tyr632fs)	KIX	PVS1, PM2	not tested	1,8	Columella below alae nasi, highly arched palate, broad thumbs and halluces, Intellectual disability, postnatal growth retardation	Hypertricho sis	Flat forehead, short neck	This work
c.1911del, p.(Asp639fs)	KIX	PVS1, PM2	not tested	6,7	No data	No data	Query Rubinstein–Taybi syndrome	This work
c.2053_2054dup, p.(Leu685fs)		PVS1, PM2	not tested	1,5	No data	No data	Query Rubinstein–Taybi syndrome	This work
c.2429dup ,p.(Met810fs)		PVS1, PM2	not tested	7,4	Microcephaly, downslanted palpebral fissures,	No data	Strabismus, long eyelashes, supernumerary	This work

					highly arched eyebrow, convex nasal ridge, columella below alae nasi, highly arched palate, broad thumbs and halluces, Intellectual disability, postnatal growth retardation		tooth, low anterior hairline	
c.2663del, p.(P888fs)		PVS1, PM2	not tested	1,2	No data	No data	Query Rubinstein–Taybi syndrome	This work
c.2898del, p.(Ser967fs)		PVS1, PM2	not tested	4,7	No data	No data	Query Rubinstein–Taybi syndrome	This work
c.3392dup, p.(Asn1131fs)	BRD	PVS1, PS2, PM2	de novo	0,9	Microcephaly, downslanted palpebral fissures, highly arched eyebrow, convex nasal ridge, columella below alae nasi, highly arched palate, broad thumbs and halluces, Intellectual disability,	No data	Dacryocystitis, facial hemangioma, brachidactyly	This work

					postnatal growth retardation			
c.3544_3554del, (p.Ala1182fs)	BRD	PVS1, PM2	not tested	10,1	No data	No data	Query Rubinstein–Taybi syndrome	This work
c.4074del, p.(Phe1358fs)	HAT	PVS1, PM2	not tested	4,2	Microcephaly, downslanted palpebral fissures, highly arched eyebrow, convex nasal ridge, highly arched palate, typical smile, broad angulated thumbs, broad halluces, Intellectual disability, postnatal growth retardation	No data	Strabismis, cryptorchidism	This work
c.4129_4132dup, p.(Arg1378fs)	HAT	PVS1, PS2, PM2	de novo	1,1	No data	No data	Query Rubinstein–Taybi syndrome	This work
c.4729_4733del, p.(Gly1577fs)	HAT	PVS1, PM2	not tested	No data	No data	No data	Query Rubinstein–Taybi syndrome	This work

c.5757_5769dup, p.(Val1924fs)		PVS1, PM2	not tested	0,9	Microcephaly, highly arched eyebrow, columella below alae nasi, broad thumbs, halluces and distal phalanx of other fingers, Intellectual disability, postnatal growth retardation		Strabismus, hypermetropia, epicanthal folds thin lips, long philtrum, low-set ears, 2-3-4-5 finger cutaneous syndactyly and 1-2-3 toe syndactyly, short thorax, cardiovascular anomalies (bicuspid aortic valve, mild aortic valve stenosis)	This work
**Nonsense variants**								
c.316C>T, p.(Gln106*)		PVS1, PM2	not tested	6,2	Microcephaly, downslanted palpebral fissures, highly arched eyebrow, columella below alae nasi, highly arched palate, broad thumbs, halluces and distal phalanx of other fingers, Intellectual disability,	No data	Synophrys, overhanging nasal tip, long eyelashes, strabismus, epicanthal folds, dysplastic ears, auricular pit, short neck, scoliosis, cryptorchidism	This work

					postnatal growth retardation			
c.445C>T, p.(Gln149*)		PVS1, PM2	not tested	0,2	No data	No data	Query Rubinstein–Taybi syndrome	This work
c.733C>T, p.(Gln245*)		PVS1, PM2	not tested	No data	No data	No data	Query Rubinstein–Taybi syndrome	This work
c.1063C>T, p.(Gln355*)	TAZ1	PVS1, PM2	not tested	0,2	No data	No data	Query Rubinstein–Taybi syndrome	This work
c.1114C>T, p.(Gln372*)	TAZ1	PVS1, PM2	not tested	0,7	No data	No data	Query Rubinstein–Taybi syndrome	This work
c.1270C>T, p.(Arg424*)	TAZ1	Previously described as pathogenic	not tested	12,5	No data	No data	Query Rubinstein–Taybi syndrome	CM053181, PMID: 16021471, 32827181
c.1447C>T, p.(Arg483*)		Previously described as pathogenic	not tested	2	No data	No data	Query Rubinstein–Taybi syndrome	CM1918549, PMID: 31566936
c.1447C>T, p.(Arg483*)		Previously described as pathogenic	not tested	0,1	No data	No data	Query Rubinstein–Taybi syndrome	CM1918549, PMID: 31566936

c.1522C>T, p.(Gln508*)		PVS1, PM2	not tested	0,1	No data	No data	Query Rubinstein–Taybi syndrome	This work
c.2218G>T, p.(Gly740*)		PVS1, PM2	not tested	10,1	Microcephaly, highly arched eyebrow, columella below alae nasi, mild intellectual disability, postnatal growth retardation	Hypertrichosis	Obesity, absent speech, strabismus, epicanthal folds, low anterior hairline, overhanging nasal tip, cryptorchidism	This work
c.3441C>A, p.(Tyr1147*)	BRD	PVS1, PM2	not tested	1,2	Downslanted palpebral fissures, highly arched eyebrow, broad thumbs, halluces and distal phalanx of other fingers, Intellectual disability, postnatal growth retardation	Hypertrichosis of shoulders	Long philtrum, low-set dysplastic ears, short neck, low anterior hairline	This work
c.3690T>G, p.(Tyr1230*)		Previously described as pathogenic	not tested	0,5	Microcephaly, downslanted palpebral fissures, highly arched eyebrow, convex nasal ridge,	Hypertrichosis	Cryptorchidism, caliectasis, low anterior hairline, strabismus, epicanthal folds	CM2023000, PMID: 32827181

					columella below alae nasi, highly arched palate, broad thumbs and halluces, Intellectual disability, postnatal growth retardation			
c.3911C>A, p.(Ser1304*)	PHD	PVS1, PM2	not tested	1,2	Microcephaly, downslanted palpebral fissures, highly arched eyebrow, broad thumbs and halluces, Intellectual disability, postnatal growth retardation	No data	Low-set ears, short nose with overhanging nasal tip, duplicated tongue tip, inguinal hernia	This work
c.4380T>A, p.(Tyr1460*)	HAT	PVS1, PM2	not tested	2	Downslanted palpebral fissures, highly arched palate, broad thumbs, duplicated halluces, Intellectual disability,	No data	Neck pterygium, hypoplastic teeth	This work

					postnatal growth retardation			
**Splicing variants**								
c.1574-2A>G		PVS1, PM2	not tested	3,6	No data	No data	Query Rubinstein–Taybi syndrome	This work
c.1824-1G>C		PVS1, PM2	not tested	4,4	Downslanted palpebral fissures, convex nasal ridge, columella below alae nasi, broad thumbs and halluces, Intellectual disability, postnatal growth retardation	Hypertrichosis	Epicanthal folds, cryptorchidism	This work
c.3242_3250+6del		PVS1, PM2	not tested	No data	No data	No data	Query Rubinstein–Taybi syndrome	This work
c.3609+1G>A		Previously described as pathogenic	not tested	1,2	Microcephaly, downslanted palpebral fissures, highly arched eyebrow, columella below alae nasi, broad thumbs and	No data	Obesity, hypotonia	CS2057719, PMID: 33057194, 35982159

					halluces, Intellectual disability, postnatal growth retardation			
c.3610-1G>C		PVS1, PM2	not tested	9,6	No data	No data	Query Rubinstein–Taybi syndrome	This work
c.3698+5G>A		PS1, PS2, PM2	de novo	6,6	highly arched palate, broad thumbs and halluces, Intellectual disability	Hypertrichosis	Hypotonia, conductive deafness, left ear canal atresia, narrow forehead, gastroesophageal reflux, diaphragmatic hernia, constipation	This work
c.4279_4280+1del		PVS1, PM2	not tested	2,8	No data	No data	Query Rubinstein–Taybi syndrome	This work
c.4560+1G>A		PVS1, PM2	not tested	14	Microcephaly, downslanted palpebral fissures, highly arched eyebrow, columella below alae nasi, broad thumbs and halluces, Intellectual	Hypertrichosis	Obesity, scoliosis, strabismus, epicanthal folds, hypermetropia, low anterior hairline	This work

					disability, postnatal growth retardation			
**Missense variants**								
c.4340C>T, p.(Thr1447Ile)	HAT	Previously described as pathogenic	not tested	7	Microcephaly, convex nasal ridge, downslanted palpebral fissures, Intellectual disability, postnatal growth retardation	Hypertrichosis	Normal thumbs and halluces, brachydactyly, cutaneous syndactyly of the 2nd and 3rd toes	CM050568, PMID: 15706485
c.4439A>G, p.(Asp1480Gly)	HAT	Previously described as pathogenic	not tested	4	Broad thumbs and halluces, narrow palate, convex nasal ridge; intellectual disability, postnatal growth retardation	Hypertrichosis	Mild upslanted palpebral fissures, long eyelashes, ptosis, micrognathia, 2-3 toes cutaneous syndactyly, postaxial polydactyly of the left hand, atresia of the nasolacrimal duct, inguinal and umbilical hernia	CM1925364 PMID: 31216405, 32827181, 33057194

c.4439A>G, p.(Asp1480Gly)	HAT	Previously described as pathogenic	not tested	3,2	No data	No data	Syndromic neurodevelopmental disorder, query Rubinstein–Taybi syndrome	CM1925364 PMID: 31216405, 32827181, 33057194
EP300 (NM_001429.4)								
**Frameshift variants**								
c.4043dup, p.(Met1349fs)	HAT	PVS1, PM2	not tested	14,3	Microcephaly, (dismorfic facial features?), broad thumbs, duplicated halluces, moderate intellectual disability, postnatal growth retardation	No data	cryptorchidism	This work
**Nonsense variants**								
c.1459C>T, p.(Gln487*)		PVS1, PM2	not tested	8,9	Microcephaly, convex nasal ridge, highly arched palate, broad thumbs and	No data	External genital hypoplasia	This work

					halluces, mild intellectual disability, postnatal growth retardation			
c.4384C>T, p.(Arg1462*)	HAT	Previously described as pathogenic	not tested	1,6	Microcephaly, highly arched eyebrow, broad thumbs and halluces, delayed development, postnatal growth retardation	Maternal pre-eclampsia	Broad nasal tip, epicanthal folds	CM2119308, PMID: 34427995, 35616356
**Splicing variants**								
c.1878_1878+1del	KIX	PVS1, PM2	not tested	5,6	No data	No data	Query Rubinstein–Taybi syndrome	This work
SRCAP (NM_006662.3)								
c.7303C>T, p.(Arg2435*)		Previously described as pathogenic	not tested	11,9	No data	No data	Query Rubinstein–Taybi syndrome	CM121173, PMID: 22265015, 38230957
**VoUS variants**								

CREBBP								
**Splicing?**								
c.1941+5G>A	KIX	PM2, PP3	not tested	0,8	No data	No data	Query Rubinstein–Taybi syndrome	This work
**Missense**								
c.3698G>C, p.(Arg1233Thr)		PM2, PP3	not tested	5,5	No data	No data	Query Rubinstein–Taybi syndrome	This work
c.5486A>T, p.(His1829Leu)	TAZ2	PM2, PP3	not tested	No data	No data	No data	Query Rubinstein–Taybi syndrome	This work
**Inframe**								
c.4240_4254del p.(Val1414_Gly1418del)	HAT	PM2, PP3	not tested	6,5	No data	No data	Gait abnormality, phenotype of RSTS	This work
EP300								
c.3749G>A, p.(Cys1250Tyr)	PHD	PM2, PP3	not tested	4,1	Microcephaly, convex nasal ridge, columella	Maternal pre-eclampsia,	Strabismus, thin lips, enamel hypoplasia, carious teeth,	This work

					below alae nasi, highly arched palate, broad thumbs and halluces, intellectual disability	hypertrichosis	ventricular noncompaction	
